# Fly-by-Feel: Advancements and Applications of Bio-Inspired Wind-Hair Sensors on Fixed-Wing UAVs

**DOI:** 10.3390/biomimetics11070500

**Published:** 2026-07-16

**Authors:** Omar Selim, Alecsandra Court, Christoph Brücker

**Affiliations:** Department of Engineering, City St. George’s, University of London, Northampton Square, London EC1V 0HB, UK; alecsandra.court.2@citystgeorges.ac.uk (A.C.); christoph.bruecker@citystgeorges.ac.uk (C.B.)

**Keywords:** biomimetics, fly-by-feel, bio-inspired sensing, distributed flow sensing, UAV, stall detection, aerodynamic sensing

## Abstract

Distributed aerodynamic sensing is a key requirement for future fly-by-feel UAV systems. Inspired by mechanosensory systems found in flying animals, this paper investigates the use of a bio-inspired optically tracked flexible pillar sensor array for aerodynamic sensing and stall detection on a washed-out NACA0012 aerofoil. Experiments were conducted in a low-speed water tunnel, with flow at chord-based Reynolds $Re=70\times 10^{3}$ and the pillar sensors set to measure local flow conditions. Sensor calibration and dynamic characterisation were performed prior to testing. Time-resolved flow visualisation measurements were used to validate sensor response and investigate local flow phenomena. The results demonstrated that flexible pillar sensors can capture early indications of stall through monitoring of spanwise mean deflection, flow reversal events associated with incipient and fully separated flow, and characteristic low-frequency oscillations. The findings demonstrate the potential of distributed bio-inspired sensor arrays to enhance stall detection and enable real-time aerodynamic monitoring in future fly-by-feel UAV systems.

## 1. Introduction

Natural fliers rely extensively on distributed mechanosensory systems to perceive local aerodynamic conditions during flight. These systems frequently employ compliant sensing structures whose deformation under aerodynamic loading provides continuous information regarding the surrounding flow environment. Inspired by such biological sensing architectures, biomimetic adaptations of such sensing principles are seen in engineered systems such as within strain gauges, load cells, and pressure sensors, where flexible elements embedded within the systems to deform due to loads are essential to sense and report on the intended quantities. In practice, the known material properties of a certain element allow a repeatable and deterministic response to stimulus, which is subsequently used to quantify the magnitude and direction of the stimulus, whether arising from bodily interactions such as applied forces or dynamic factors like fluid velocity.

Flexible element sensors are commonly found present in a variety of flying animal species through different domains. Particularly on the wings of bats, it is seen that the wings are covered with rows of microscopic flexible wind-hairs arranged in grids [1,2], which serve a mechanoreceptor purpose to inform flight control. Earlier work investigated the mechanoreceptors in covert feathers of various bird species which were also seen to provide sensory information to aid flight efficiency and stability [3,4]. These same principles are observed in insect flight, with the difference of scales and sensor types [5,6], and it was found in [7] that such sensors have capabilities to add the sensing of structural geometric changes within the wing in addition to flow information, further informing the system. Mechanosensation in flying animals typically requires not only the capability to sense mechanical forces themselves [8], but also to be able to ascertain trends in differential forces from the baseline [9]. An example of this is seen particularly in fast-flying birds of prey. It was seen in Peregrine falcons that there exist specific mechanoreceptor dorsal feathers responsible for sensing changes in the frequency domain which inform the bird on flight attitudes and angle of attack [10], an important flight characteristic which allows the bird to fly at marginal stability to achieve superior agility and maneuverability in hunting [11].

The development of the sensors discussed herein was inspired by the nature of biological sensing to be a distributed, robust, and redundant system of sensors, typically providing multimodal environmental information [12]. The design and development and the results of preliminary tests in air are discussed in [13,14] where it is shown how a single row of sensors placed at specific locations and patterns can provide spatially distributed aerodynamic information toward enabling fly-by-feel flow-state awareness. The flexible pillar sensors were designed and fabricated in-house and are identical to those developed and characterised in [13,14]. The sensors do not transmit signals through internal wiring but, instead, the fluorescent-coated tips are tracked optically via wavelength-filtered high-speed imaging of tip deflection, enabling remote, distributed non-intrusive flow-state sensing. Careful in situ calibration and dynamic testing was carried out in the studies included herein which were similar to the methods outlined in [14]. Underwater tests monitoring the deflection of whiskers using similar techniques as those used within this study are shown in [15] where a deep learning algorithm was developed and integrated to locate upstream obstacles. Optical monitoring of flexible structures was shown in [16] where similar optically monitored elastic sensors were used to quantify the wall shear stress in an aortic heart valve model, demonstrating the versatility provided by the use of biologically inspired systems with distributed compliant, non-contact, optical sensing. The relative size of the sensors dictates the sensitivity to changes in the local boundary layer characteristics [17], which allows the monitoring of the spatial evolution of the boundary layer over various geometries. Recent advances in bio-inspired aerodynamic sensing have highlighted the growing potential of distributed flow-sensitive mechanoreceptive systems for flight-state awareness and flow event detection [18]. Unlike distributed biological sensing systems, conventional UAV aerodynamic sensing frequently relies on isolated point measurements, whereas more advanced systems such as laser Doppler velocimetry (LDV) can provide highly precise flow measurement [19], particularly for physically restrictive geometrical constraints. However, these require coherent light sources, sensitive alignment, and are costly and impractical for small UAV deployment.

Previous work on distributed strain and airflow sensing for UAV flight control has similarly demonstrated the advantages of distributed aerodynamic sensing over conventional rigid-body state estimation approaches [20]. The approach presented herein explores distributed surface-based aerodynamic sensing through passive compliant sensing elements and low-cost imaging, toward enabling distributed fly-by-feel aerodynamic perception for UAV systems.

**Figure 1:**
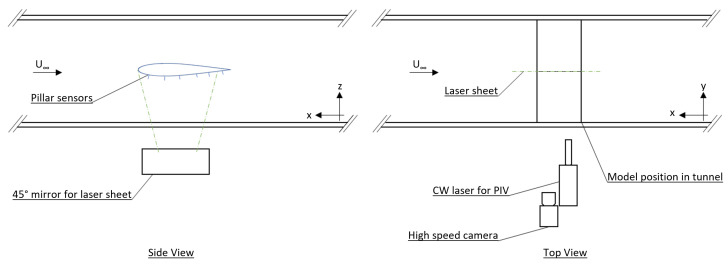
Water tunnel experimental setup for PIV experiment.

Herein, the results from tests of a flexible element sensor developed to provide distributed flow measurements in various single-phase flows are presented and analysed. The novelty of this study is showcasing the concept of implementing real-time distributed detection of stall-related flow phenomena using an optically tracked flexible pillar sensor array on an aerofoil. The tests presented are intended to expand on the results from [14], where a single row of flexible sensors was tested in a wind tunnel on an NACA0012 symmetric aerofoil, a widely used benchmark geometry in aerodynamic research, to determine their capability to perceive evolving aerodynamic conditions and their ability to characterise specific pre-stall phenomena. The NACA0012 aerofoil was deliberately chosen as a canonical, well-documented profile to allow comparison with a wide body of existing literature and to isolate stall-related flow features in a controlled setting. While the simplicity of the geometry limits generalisation, this study serves as a foundational step toward future bio-inspired aerodynamic sensing and adaptive flight control systems. In particular, the tests reported herein were carried out in a water-tunnel environment (Figure 1) in an effort to achieve the following objectives. Primarily, the water-tunnel tests were carried out to facilitate high-resolution flow visualisation and time-resolved PIV measurements while maintaining chord-based Reynolds numbers representative of small- to medium-scale UAV flight conditions. The lower flow velocities achievable in water enabled detailed observation of the interaction between the distributed pillar array and the surrounding flow field. The conditions for the tests are discussed in detail further in the paper and the differences are highlighted between the underwater tests and tests carried out in air, particularly on the dynamic response of the pillar sensors. Tests were also carried out to measure the distributed sensor interaction effects, in addition to examining the results of arranging the sensors in an array, exploring their response to localised aerodynamic disturbances, such as stall or high turbulence, on a washed-out wing.

## 2. Materialsand Methods

The results reported herein are of the experimental work carried out in City St. George’s University low-speed water tunnel shown in Figure 1. The water tunnel is an open-surface, closed loop tunnel, with a transparent test section of 40 × 50 × 120 cm which allows good optical access for both the PIV experiments and the sensor tracking experiments carried out.

### 2.1. PIV Setup

The model was setup to span the width of the tunnel, such that the span axis is parallel to the open surface. A continuous wave Argon-Ion laser (Raypower 5000, Dantec Dynamics, Skovlunde, Denmark, 5 W power at λ = 532 nm) was used as an illumination source aligned with a high-speed camera as shown in (Figure 1). The laser source was positioned in line with and below the camera. A $45^{\circ }$ mirror was placed beneath the tunnel to reorient the beam by $90^{\circ }$, producing a laser sheet orthogonal to the camera’s optical axis, as illustrated in the figure.

Neutrally buoyant particles (hollow glass spheres, diameter 50 μm) were mixed into the fluid and allowed to homogenise before measurements were taken. These seeding particles were introduced specifically for the PIV measurements rather than relying on naturally occurring particles present in the water, which has typically yielded poor correlation due to their irregular shape, variable size, and lack of neutral buoyancy. The controlled use of uniformly shaped particles ensured reliable and accurate reconstruction of the velocity field. The resulting seeding concentration produced an average of 7 particles per 32 × 32 pixel interrogation window, consistent with prior work using the same setup.

A Phantom M310 high-speed camera (Adept Turnkey, Perth, Australia) was arranged orthogonally to the laser sheet and fitted with a varifocal lens used at focal lengths of 100 mm and 300 mm for wide-angle and close-in measurements, respectively. The laser sheet thickness was approximately 1 mm. Image sequences were captured at 3000 fps, but every other frame was used in processing, resulting in an effective inter-frame time (Δt) of 0.666 ms. This provided a mean particle image displacement of approximately 1.94 pixels per frame.

The field-of-view magnification factor was 0.06. Image processing was performed using PIVLab [21], employing a standard multi-pass cross-correlation algorithm with sub-pixel Gaussian fitting yielding a velocity uncertainty on the order of $1\times 10^{-3}$ m/s, with sub-pixel displacement uncertainty estimated between 0.03 and 0.05 pixels. The first pass used a 64 × 64 pixels interrogation window, followed by three refinement passes at 32 × 32 pixels with a slight reduction to 30 pixels in the final pass. A 50% overlap (32 pixels step size) was applied. Outlier removal was performed using a standard deviation filter of 8, and a median filter of 3. The calibration factor was determined as 1 pixels = 0.0001 m.

### 2.2. Sensing Wing Preparation

Work already carried out at City, University of London produced flexible pillar sensors [14], which act as a bio-inspired surface-mounted sensor array when applied to an aerofoil, representing arrays of biomimetic wind-hairs that sense the flow. The deflection of the pillars scale with the local Reynolds number, as described in [14], and therefore by employing the pillar sensors within an array along the suction side of the wing, the effect of aerodynamic phenomena on local flow can be detected and quantified.

**Figure 2:**
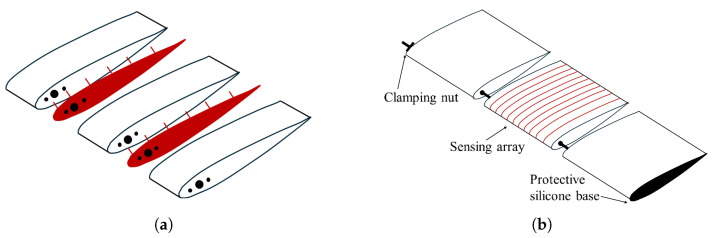
Illustration of the sensor integration into the aerofoil test model. Not to scale. (**a**) Exploded view sketch of two hair sections clamped between 3D-printed aerofoil sections. (**b**) Sketch of the full sensing model with top and bottom aerofoil sections clamping the sensor array.

**Figure 3:**
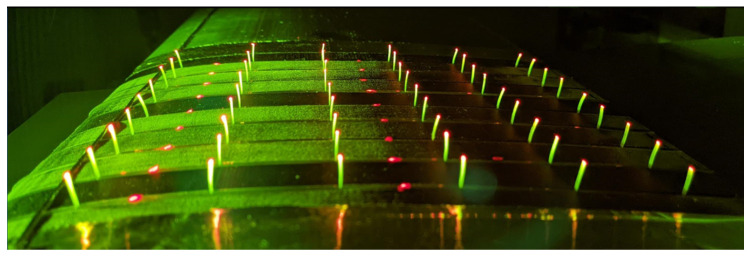
View of the suction side of the sensing wing under the LED light sheet and filter in flow-off condition. All 9 rows of pillars visible. Flow would be left to right.

**Figure 4:**
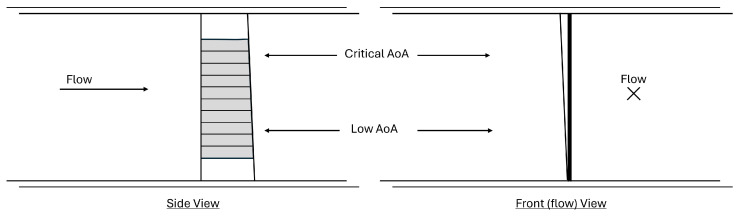
Water tunnel experimental setup for pillar array experiment—figure illustrates the washout within the section design—dimensions illustrative and not to scale.

The sensor array was embedded on the suction side of an NACA0012 wing section, as shown in Figure 2 and Figure 3. The pillars have the same dimensions as those used in [14], with a rectangular cross-section measuring 1.5 mm in the spanwise direction and 0.3 mm in the chordwise direction. The array consists of 6 pillars arranged chordwise at 15% chord spacing starting at 15% chord, and 9 rows spaced with 8.6% chord spanwise, forming a 6×9 grid of sensors. The wing incorporated a designed washout (twist) across the span. As a result, the local angle of attack at the root (top section) of the wing was higher than that at the tip (lower section), as illustrated in Figure 4. The wing was placed and adjusted such that the angle of attack experienced by the inboard row of sensors was at a critical pre-stall condition at $11^{\circ }$ angle of attack, with the angle gradually decreasing along the span towards the tip such that the outboard row was at $8^{\circ }$. This setup was based on known aerofoil characteristics and previous test results, and was validated through preliminary TR-PIV measurements conducted before the main array experiments.

The selected condition provided a controlled spanwise variation in local flow state, placing the inboard section near incipient stall while maintaining attached flow over the outboard section. This enabled the simultaneous observation of attached and pre-stall flow conditions across the sensor array and was considered particularly suitable for investigating boundary layer growth, flow separation, and stall-related phenomena using distributed bio-inspired sensing.

The same optical setup described in Figure 1 was used, replacing the laser sheet with a wider LED illuminator (IL-105/6X Illuminator, HardSoft, Obergriesbach, Germany) to enhance illumination of the pillar tips.The fluorescent tips were captured at a sampling rate of 1000 Hz and processed using a modified version of an in-house MATLAB-based cross-correlation algorithm (MATLAB Release R2023b), which compares small interrogation windows around the pillar tips to their wind-off position, enabling simultaneous tracking of the full sensor array. The details of this algorithm are further discussed in [14].

The pillars are laser cut from silicone sheets of thickness 1.5 mm and density 1.2 g/cm^3^. Each cut section is an NACA0012 aerofoil cross-section of chord 20 cm and each has 6 pillars at even spacing of 15% chord along the suction side. These sections are then clamped in between 3D-printed aerofoil sections to produce a modular sensing aerofoil, where the pillars act as cantilever beams. The pillars have a rectangular cross-section of 1.5 mm spanwise by 0.3 mm chordwise; these dimensions were chosen to emphasise deflection in the chordwise direction thus providing a clear instantaneous flow picture, with reduced noise from other out of plane motion. The material properties and setup are summarised in Table 1 and the clamping mechanism is illustrated in Figure 2.

### 2.3. Reynolds Number Scaling and UAV Relevance

The experiments were conducted at a chord-based Reynolds number of $Re_{c}$ = 70,000, corresponding to flow conditions representative of the lower end of the flight envelope encountered by small- to medium-scale fixed-wing UAVs, such as launch and recovery, where stall phenomena are more prevalent. The use of a water tunnel enabled these Reynolds numbers to be achieved at substantially lower free-stream velocities than would be required in air, facilitating high-resolution flow visualisation and time-resolved PIV measurements.

The objective of the study was to investigate the ability of distributed flexible pillar sensors to detect flow-state signatures associated with boundary-layer growth, flow separation, flow reversal, and stall onset. These phenomena are governed primarily by the local flow state and Reynolds number. Consequently, the water-tunnel environment provides a suitable platform for investigating the sensing principles considered herein while maintaining flow conditions relevant to small- and medium-scale UAV applications.

### 2.4. Sensor Calibration

The sensor calibration process for the water-tunnel experiment involved an impulse excitation test on one of the leading edge pillars to determine the physical parameters of the submerged pillars. To establish the velocity calibration, the free-stream velocity was increased incrementally and pillar recordings were taken in the same method as before, and mean values obtained. These were compared to free-stream Particle Image Velocimetry (PIV) measurements to construct a comprehensive velocity curve. This calibration methodology draws parallels to previous work outlined in [13], with the distinct difference that the velocity in this study is the free-stream velocity, whilst in the previous studies, it was the local velocity. The velocity increments covered the entire range of experimental conditions.

**Figure 5:**
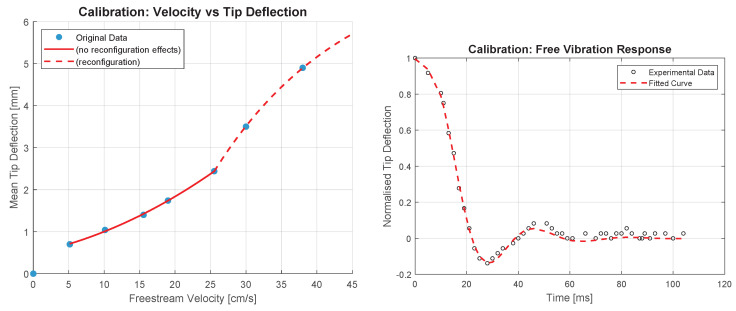
Sensor calibration results. (**left**) Tip deflection under controlled flow for velocity sensitivity calibration. (**right**) Time-domain response used to determine the structural eigenfrequency.

The velocity calibration shown in Figure 5 was represented using separate fitted relationships below and above the onset of pillar reconfiguration. Prior to reconfiguration, the mean tip deflection was well described by a second-order polynomial fit, while an exponential asymptotic fit was employed above the reconfiguration threshold to account for the reduced sensitivity as the pillar approached its maximum operable deflection. The resulting calibration relation is given by

(1) $$Q\left(v\right)=\left\{\begin{array}{cc} 0.0016v^{2}+0.0350v+0.4890, & v\leq v_{r},\\ 7.50-5.07\exp\left[-0.053(v-25.52)\right], & v>v_{r}, \end{array}\right.$$

where *Q* is the mean tip deflection in mm, *v* is the free-stream velocity in $\mathrm{cm}\;s^{-1}$, and $v_{r}\approx 25\;\mathrm{cm}\;s^{-1}$ denotes the onset of reconfiguration. The optical tracking uncertainty was estimated as 0.03–0.05 pixels based on the cross-correlation methodology employed [22] corresponding to a tip-deflection uncertainty of approximately 0.01 mm which yields an estimated velocity uncertainty below 0.1 cm s^−1^ in the most sensitive operating regime. In the low-velocity regime, the sensor exhibited an approximately linear response of $0.136\;\mathrm{mm}\;{\left(\mathrm{cm}\;s^{-1}\right)}^{-1}$.

Furthermore, dynamic parameters such as natural frequency and damping coefficients were determined through an impulse excitation test, where the pillar was manually deflected to its maximum operable displacement and released, allowing the free-decay motion to be optically tracked. The results of these tests are illustrated in Figure 5. The first plot shows the relationship between mean tip deflection and free-stream velocity, while the second plot illustrates the normalised damped oscillatory response of a submerged sensor following release from deflection. The purpose of the impulse excitation test was to identify the dominant dynamic response of the sensor. Previous structural analyses of the same pillar geometry reported a dominant first bending mode with substantial frequency separation from higher-order bending and torsional modes [14]. Although fluid loading in the present submerged configuration reduced the measured natural frequency, the free-decay response was found to be well described by a single underdamped oscillatory mode. Consequently, a linear underdamped second-order representation was considered sufficient for dynamic characterisation. A second-order underdamped model was therefore fitted to the raw free-decay deflection signal, independently of the low-pass filtering procedure later applied to the flow-measurement time histories. The oscillatory response was modelled as

(2) $$x\left(t\right)=Ae^{-ct}\cos\left(\omega t+\phi \right)+B$$

where *A* is the initial amplitude, *c* is the decay rate, ω is the damped natural frequency, ϕ is the phase offset, and *B* is a baseline shift. Fitting this model to the normalized deflection signal yielded a damped natural frequency of approximately $f_{d}=18.7\;\mathrm{Hz}$, with a decay rate $c=74.9\;s^{-1}$, and an estimated damping ratio:

(3) $$\zeta =\frac{c}{\omega_{n}}\approx 0.54$$

The parameters of Equation (2) were estimated using nonlinear least-squares fitting in MATLAB. The measured free-decay response was normalised by the maximum measured tip deflection, and the model parameters were obtained by minimising the residual error between the measured and fitted deflection. The identified damped natural frequency and damping ratio provide a practical assessment of the sensor dynamics relevant to the present study. The response settled to near-zero amplitude within approximately 60 ms, consistent with the identified second-order dynamics. Since the aerodynamic analysis presented herein focuses on low-frequency flow phenomena below the 5 Hz filtering threshold, the frequencies of interest remain well separated from the dominant structural response of the sensor. These results went on to inform the signal processing and filtration strategy outlined in Section 3.

## 3. Results

### 3.1. Leading Edge PIV

TR-PIV tests were carried out to investigate further the flow immediately about the leading edge pillar with the aim of ascertaining the effect that the boundary layer may have on the leading edge pillar in addition to the effect that a pillar has on the subsequent pillar.

**Figure 6:**
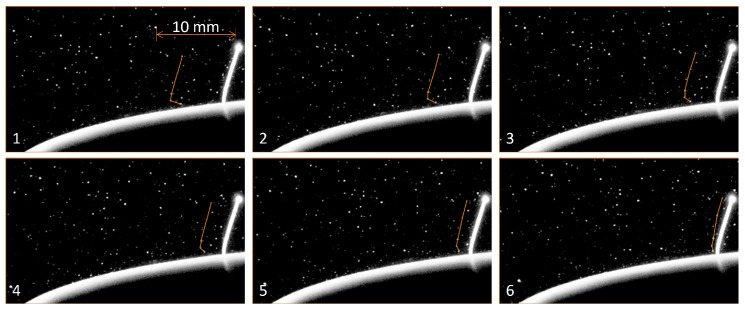
LEBL: Sequential close-up images illustrating the boundary layer decelerated flow upstream of the leading edge pillar. Flow is moving left to right with 1 being earliest capture and 6 being latest capture.

Figure 6 illustrates the retarded flow within the boundary layer in contrast to the flow outside of the boundary layer encountered by the leading edge pillar. Notably, this shows the relative uniformity of the flow experienced by the leading edge pillar and that the boundary layer thickness at that point has a minimal effect on the total pillar deflection, which is majorly affected by the flow outside of the boundary layer.

The mean tip deflection of the pillar, which is dictated by the moment balance about the clamped end of the beam M(t), is defined in [14] and given in Equation (4), where g(t,l) is the instantaneous load distribution acting normal to the longitudinal axis *l* of the pillar. This expression shows that the velocity profile along the length of the pillar (and thus the boundary layer thickness) informs the total deflection and that the effect of this profile variation is more pronounced closer to the tip than the root.

(4) $$M(t)=\int_{0}^{l}g(t,l)\times l\;dl$$

To complement this, boundary layer thickness values at the location of the first pillar for $\alpha =0^{\circ }$, $8^{\circ }$, and $11^{\circ }$ were obtained using xfoil [23]. The results showed a boundary layer thickness of 0.437 mm (6% of the pillar length) at 0°, 1.84 mm (25%) at 8°, and 5.04 mm (67%) at 11°, demonstrating the substantial increase in δ near stall. Direct quantification of how this growth affects deflection is planned as part of future wind-tunnel experiments.

**Figure 7:**
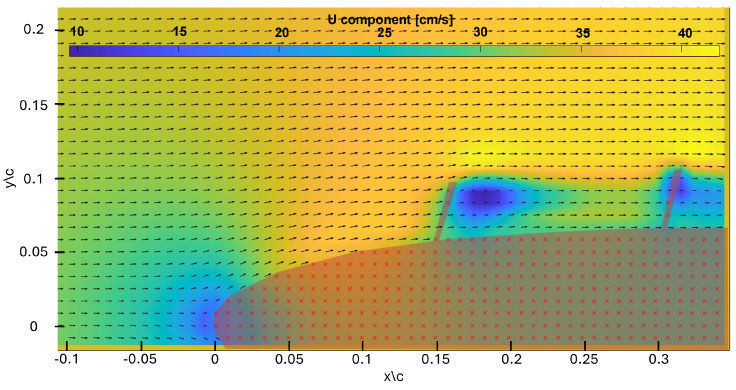
TR-PIV results taken around the leading edge of the pillars illustrating the area of decelerated flow aft of the pillar and the recovery.

**Figure 8:**
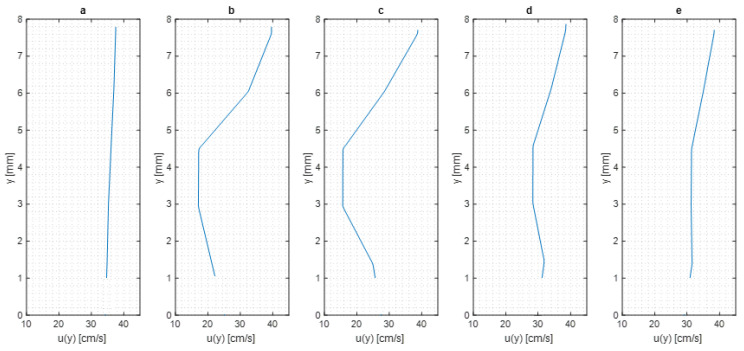
Profiles of horizontal velocity component u(y) taken at vertical profiles at locations along with (**a**) immediately upstream of the leading edge pillar and (**b**–**e**) at approx. 5 pillar diameter increments downstream of the first pillar.

In addition, the effect that the leading edge pillar has on proceeding pillars was investigated from TR-PIV results shown in Figure 7 by taking a vertical mean velocity profile of the order of a single pillar at a position upstream (Figure 8a) and 4 positions downstream (Figure 8b–e) at 5, 10, 15, and 19 pillar diameters downstream. It should be noted that the pillars are spaced at 20 major diameters apart, which means that the last profile is the one immediately incident to the following pillar. At these locations, the mean velocity was observed to drop from 36 cm/s at the upstream point to 17 cm/s at both 5D and 10D (a 53% velocity deficit), recovering to 28 cm/s at 15D (22% deficit) and 32 cm/s at 19D (11% deficit). There is a clear retardation in the mean flow downstream of the pillar due to the pillar’s shape in addition to a bulk reduction in velocity which can be attributed to the deceleration of the flow aft of the suction peak of the aerofoil. This retardation is seen to diminish over the spacing. However, the flow does not reset completely at the point immediately incident to the subsequent pillar, and some residual effect on the mean flow from the upstream pillar remains.

### 3.2. Stall Precursor Instabilities

**Figure 9:**
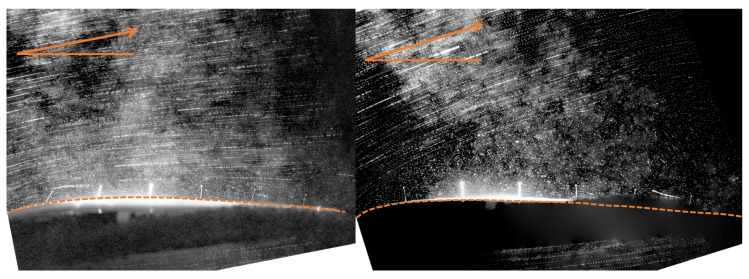
Blended images of attached pre-stall flow in comparison to separated post-stall flow and the interaction with the pillars. The orange arrow indicates free-stream flow direction relative to aerofoil chord.

Stalled flow is characterised in the results by the complete reversal of the pillars as shown in Figure 9. Even at pre-stall angles of attack, where stall is approaching but flow is not yet separated, there exist instabilities which were shown as precursors to stall. Where the boundary layer flow is dominant over the length of the pillar, the part of the pillars that remains emerged in external flow is typically that which is furthest away from the base, and thus the effect of the external flow on the pillar deflection remains dominant until the entirety of the pillar is submerged in the boundary layer. Once the boundary layer detaches and complete flow reversal is observed, the pillars revert to oscillating about their wind-off positions.

**Figure 10:**
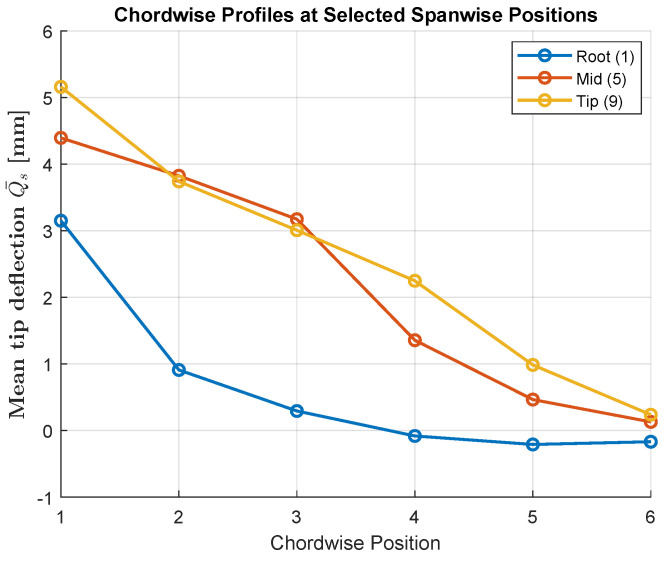
Mean tip deflection along the chordwise direction for three spanwise locations: root (position 1), mid-span (position 5), and tip (position 9).

**Figure 11:**
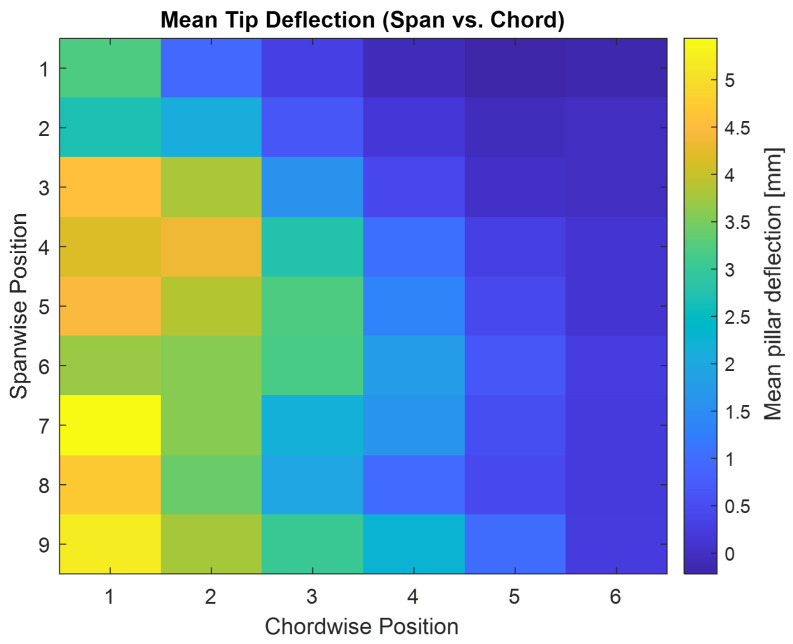
Heatmap of mean tip deflection across the 9×6 sensor array.

**Figure 12:**
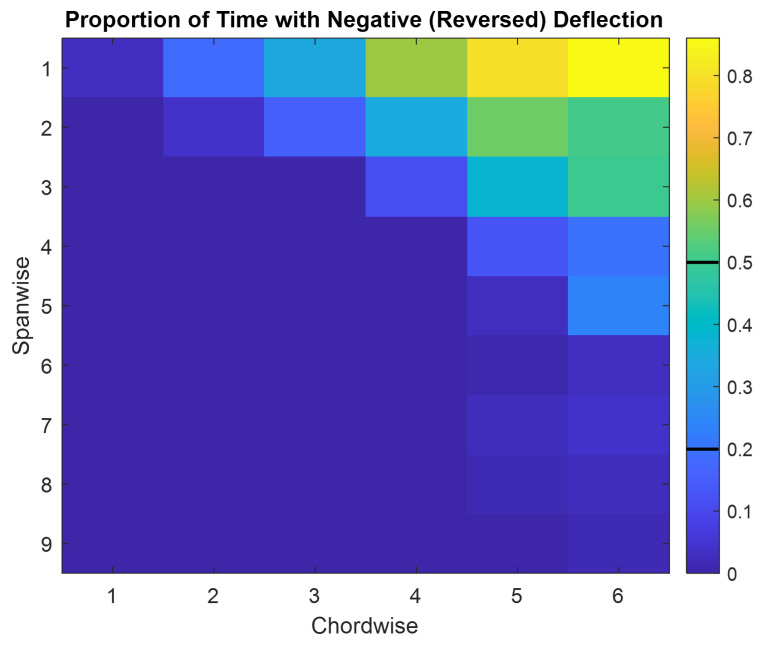
Proportion of time each sensor recorded negative tip deflection, shown as a heatmap. Regions exceeding 20% reversal (incipient separation) and 50% reversal (full separation) follow the criteria established in [14].

The tests over the array of pillar sensors were carried out at a free-stream velocity of $U_{\infty }=35$ cm/s, corresponding to a chord-based Reynolds number of $Re_{c}=70,000$. The spatial distribution of tip deflection across the sensor array reveals key indicators of flow separation and early stall behaviour, particularly in the upper spanwise region of the wing. Figure 10, Figure 11 and Figure 12 collectively demonstrate how tip deflection varies both along the chord and span, providing insight into the aerodynamic state of the flow over the wing.

The chord-wise deflection profiles in Figure 10 display the mean tip deflection for three spanwise stations at the simulated root, mid-span, and tip. The deflection is seen to reduce from the leading edge to the trailing edge in all three as expected with the chordwise boundary layer growth, but is most pronounced at the root (Span 1) where there is a steep gradient. This behaviour is consistent with the results presented in [14] where similar patterns were used to identify higher angles of attack.

Figure 11 provides a two-dimensional heatmap of mean deflection across the full 9 (spanwise) × 6 (chordwise) sensor array. The mean deflections on the upper row, corresponding to Span 1 near the root of the wing, have a reduced mean deflection compared to the mid-span and tip regions. This drop is particularly evident toward the trailing edge which suggests that the boundary layer growth in this region is sufficiently large that the free-stream component over the pillar is not producing a strong deflection. Such behaviour was seen in [14] to be characteristic of the onset of stall.

Another important marker is apparent in Figure 12 which shows the proportion of time each sensor recorded negative tip deflections. Following the findings in [14], reversal percentages exceeding 20% were taken to indicate incipient boundary layer separation, corresponding in this experiment with the trailing edge pillars between $8.5^{\circ }$ and $10^{\circ }$, while values above 50% suggested fully separated flow. These patterns can be observed in the reversal heatmap in Figure 12. The highest instances of reversal are concentrated near the upper span (AoA > $10^{\circ }$) and trailing edge where the mean deflection and gradient changes were most significant.

### 3.3. Signal Filtering

To reduce the influence of high-frequency vibrations from the sensor structure oscillating at its natural frequency, a MATLAB-based second-order zero-phase Butterworth low-pass filter was applied to the raw tip deflection time histories. The mathematical formulation and implementation details of the filter are provided in Appendix A. The cut-off frequency was set at 5 Hz, based on observations from calibration testing described in Section 2 which identified the dominant structural eigenfrequencies of the pillars as being above this threshold at 18.7 Hz as shown also in [24]. The sampling frequency of the system was 1000 Hz, ensuring the resolution to preserve the low-frequency content relevant to flow-induced oscillations. The resulting filtered tip-deflection signals were subsequently used for the calculation of mean deflection, flow-reversal statistics, and the analysis of low-frequency aerodynamic oscillations.

**Figure 13:**
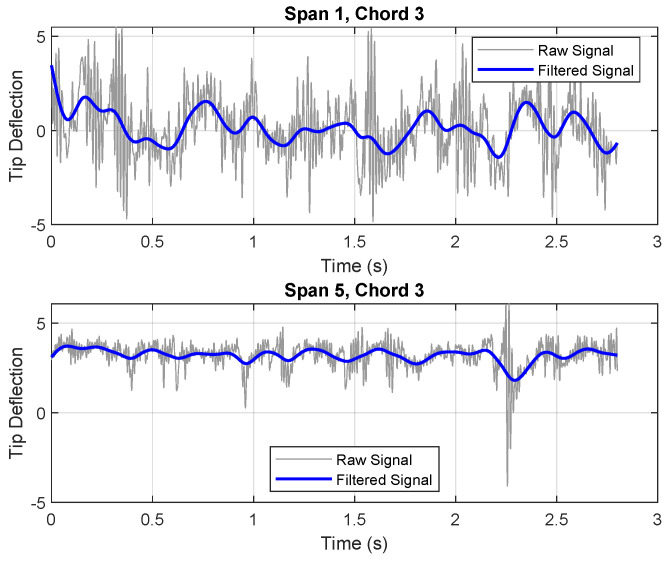
Comparison of raw (grey thin line) and low-pass-filtered (blue thick line) tip deflection signals at Span 1, Chord 3 (**top**), and Span 5, Chord 3 (**bottom**).

This filtering process was designed to attenuate non-aerodynamic noise while retaining unsteady aerodynamic features such as those associated with shear layer flapping at stall onset. The effectiveness of the filter is illustrated in Figure 13, which compares the raw and filtered signals at two representative locations on the wing. The filtered signals clearly emphasize the low-frequency aerodynamic content, while the high-frequency components—evident in the raw data—are significantly reduced. Quantitatively, the root mean square (RMS) values of the zero-mean signals were reduced from 1.69 mm to 0.84 mm at Span 1 (Chord 3), and from 0.73 mm to 0.42 mm at Span 5 (Chord 3), reflecting the suppression of structural resonance and enhanced signal clarity in regions affected by stall.

## 4. Discussion

The paper’s results provide some insights into the behaviour and capabilities of the pillar sensors in the water-tunnel test environment. The findings are discussed below along with their implications, potential applications, and limitations of the sensor system.

The calibration was carried out in situ and the sensor response fit to a second-order polynomial fit for velocities above 5 cm/s and up to velocities of 25 cm/s after which the deflection exceeds 30% of the pillar length and thus reconfiguration effects, which is the natural tendency of flexible elements to modulate their drag as they deform [25], begin to appear. For velocities higher than 25 {cm/s, an exponentially decaying curve was fit that follows the expected behaviour due to reconfiguration effects [26] similar to what was observed in wind-tunnel tests [14]. Furthermore, the impulse excitation test provided information about the sensors’ underwater damping parameters and settling time. The calibration was carried out with the purpose of properly interpreting the results from this experiment and would not be appropriate for a different environment or working fluid. Previous work in wind-tunnel experiments showed that both the dynamic and static responses from the pillars differed.

The PIV tests on the leading edge pillars revealed that the boundary layer flow contributes less than 10% of the pillar length at $0^{\circ }$ angle of attack rising to 25% at $8^{\circ }$. This indicates that the boundary layer has a negligible effect on the leading edge sensor output at low angles of attack, where that sensor’s response is primarily to bulk flow properties rather than boundary layer characteristics, and that the effects begin to take effect at moderate to high angles of attack. This suggests that further work is required to fully quantify the effect of boundary layer profile and growth on the pillar tip deflection output, in addition to possibly placing the leading edge pillar slightly upstream to reduce the effect.

Moreover, the PIV results showed flow retardation in the immediate wake of the pillar and its gradual reduction further downstream. A probe in the wake showed how the flow retardation decreased from 53% in the immediate wake to 11% 20 major diameters downstream. This suggests that sensors should be placed no fewer than 20 major diameters downstream from one another to mitigate wake-induced vibrations. The visualisations of boundary layer growth helped explain how a separated boundary layer over the sensor would subtract from the deflection and reverse the deflection past the wind-off positions in specific flow conditions. This observation serves as a marker of separated boundary layer and stall.

Tests with an array of the sensors embedded within the suction side of a washed out wing section indicated specific responses that were previously seen to be characteristic of onset flow separation [14]. These were apparent on the “inboard” section of the wing as would be expected. The observations of Figure 10, Figure 11 and Figure 12 are three indicators of reversed flow, and in a broader sense, incipient stall. The chord-wise profiles showed a characteristic change in the slope of the mean tip deflection distribution, indicating the rate of growth in the boundary layer and highlights that at the root section. The mean tip deflection heat-map further supported this by highlighting regions of diminished deflection magnitude toward the trailing edge, consistent with localized growth in boundary layer and reduction in bulk flow effect on pillar deflection. Finally, the reversal heat-map illustrated the frequency of negative deflections. This provides direct evidence of intermittent or persistent flow reversal at the inboard sections before progressing outward along the span, which was seen in [14] as a marker of incipient stall. Together, these results validate the array’s ability to detect stall development spatially and temporally.

While the use of the NACA0012 aerofoil limits the geometric complexity of the present study, it provided a necessary and well-characterised baseline for validating sensor behaviour under controlled flow separation conditions. Future work will focus on extending the sensor array testing to more complex and application-specific aerofoil geometries, including those relevant to UAV and morphing-wing platforms.

## 5. Conclusions

This paper continues investigating the application of optically tracked flexible pillar sensors in flow detection and characterisation over the wing of an NACA0012 aerofoil section, and by extension draws insights into the feasibility of using such systems in a fly-by-feel, small-to-medium-scale UAV. The results of the experiments carried out demonstrated how distributed sensing enhances a UAV’s ability to detect airflow separation, turbulence, and stall onset. Experiments using a flexible optically tracked pillar sensor array mounted on a washed-out wing clearly indicated early stall onset on the inboard section through increased deflection fluctuations, frequent flow reversal events, and characteristic low-frequency oscillations.

Compared to conventional UAV control systems that rely on sparse point sensing on the airframe, this study reinforced the advantages of distributed aerodynamic sensing architectures observed in natural fliers. Unlike conventional approaches such as pitot probes, pressure taps, or hot-wire sensors, which provide information at a limited number of discrete locations, the distributed pillar array enables simultaneous observation of flow-state development across the aircraft surface. This facilitates the simultaneous detection of a variety of flow phenomena. The results align with previous work on detecting aerofoil flow phenomena, specifically, incipient stall, using a single row of optically tracked flexible pillar sensors, while also introducing new opportunities for real-time adaptive flight control informed by distributed flow-state sensing. The paper highlighted how sensor placement and response sensitivity have an impact on the information gleaned, which had not been fully explored in prior studies. Table 2 provides a qualitative comparison between the present sensing architecture and conventional aerodynamic sensing technologies [27,28,29].

The study employed optical tracking of fluorescent tips on flexible pillar sensors, removing the need for physical wiring to each sensing element. In practical UAV applications, this could be implemented using low-cost onboard optical systems focused on key regions of interest. While the present setup used high-speed cameras with optical wavelength filtering and offline post-processing, the approach establishes a foundation for using lightweight, commercially available imaging hardware with onboard data processing. Additionally, ongoing work involving event-based cameras calibrated for flow-specific features shows promise for significantly reducing system complexity and computational overhead. While this study uses optical tracking, the approach is compatible with other methods such as piezoelectric or fibre-optic transmission, enabling future onboard integration in UAV platforms. The experimental validation presented contributes to bridging the gap between biological mechanosensation and distributed aerodynamic sensing for UAV systems.

The experiments were carried out in a controlled environment where the effects of some real-world flight scenarios might not be apparent. Specifically, the water-based tests were conducted at Reynolds numbers on the lower end of what small to medium UAVs would typically encounter in air. The increased fluid density of water also influenced the dynamic response of the pillars through added mass and damping effects, resulting in a lower measured natural frequency than previously reported for the same sensor geometry in air. This effect as discussed in Section 2 should be considered when extrapolating sensor dynamics to airborne applications. Additionally, the application of signal filtering, while effective at isolating low-frequency flow phenomena, inevitably attenuates higher-frequency content that may carry useful flow dynamics. Environmental factors such as reduced visibility, which could impair optical tracking, and conditions like icing, which could alter the mechanical response of the sensors, were also not represented in the present study.

Future work should therefore aim to run wind-tunnel experiments on more complex geometries with an integrated ’online’ processing in a more repeatable setup, in addition to implementing the sensors into a test-bed UAV and evaluating the system across a range of flight conditions and operational platforms, and to quantitatively assess the impact of the sensor array in comparison to currently deployed aerodynamic sensing hardware. Furthermore, the integration of advanced machine learning algorithms in the characterisation and interpretation of data from distributed sensor arrays [30,31,32] offers a promising path toward more adaptable and robust deployment strategies. Recent work has similarly demonstrated the capability of distributed aerodynamic sensing architectures to estimate aerodynamic state information across a wide range of flight conditions, in line with the future development of fly-by-feel UAV systems [33]. In summary, this paper demonstrates the viability of bio-inspired distributed flow sensing in UAVs, highlighting its potential to enhance stability, control, and energy efficiency.

## Figures and Tables

**Table 1:** Dimensions and spacing of flexible pillars used in the sensor array.

Parameter	Value
Cross-sectional shape	Rectangular
Width (spanwise)	1.5 mm
Thickness (chordwise)	0.30 mm
Density	1.2 g/cm^3^
Young’s Modulus	2.45 MPa
Number of chordwise pillars	6
Chordwise spacing	15% of chord
Number of spanwise rows	9
Spanwise spacing	8.6% of chord
Total number of pillars	54

**Table 2:** Qualitative comparison between the proposed flexible pillar sensor array and conventional aerodynamic sensing technologies.

Technology	Spatial Coverage	Response Speed	Integration Complexity	Distributed Stall Sensing
Pitot tube	Single point	High	Low	Low
Pressure taps	Discrete points	High	Moderate–High	Moderate
Hot-wire anemometry	Single point	Very high	High	Low
LDV/PIV	Full field	Very high	Very high	Laboratory only
Flexible pillar array	Distributed	Moderate	Moderate	High

## Data Availability

The original contributions presented in this study are included in the article. Further inquiries can be directed to the corresponding author.

## Appendix A. Signal Filtering Procedure

The raw pillar tip-deflection time histories were filtered using a second-order low-pass Butterworth filter implemented in MATLAB. The filter was designed using the built-in butter() function with a cut-off frequency of 5 Hz and a sampling frequency of 1000 Hz. Zero-phase filtering was achieved using the filtfilt() function, which applies the filter in both the forward and reverse directions to eliminate phase distortion.

The discrete filter may be represented by the transfer function [34,35]

(A1) $$H\left(z\right)=\frac{b_{0}+b_{1}z^{-1}+b_{2}z^{-2}}{1+a_{1}z^{-1}+a_{2}z^{-2}}$$

where $b_{i}$ and $a_{i}$ are the filter coefficients generated by the MATLAB filter design routine and $z^{-1}$ denotes the unit delay operator.

The filter was applied directly to the raw tip-deflection time histories. The resulting filtered signals were subsequently used for the calculation of mean tip deflection, flow-reversal statistics, and the analysis of low-frequency aerodynamic oscillations. Representative examples of the raw and filtered signals are shown in Figure 13.
